# Caroli's Disease: Current Knowledge of Its Biliary Pathogenesis Obtained from an Orthologous Rat Model

**DOI:** 10.1155/2012/107945

**Published:** 2011-07-06

**Authors:** Yasunori Sato, Xiang Shan Ren, Yasuni Nakanuma

**Affiliations:** Department of Human Pathology, Graduate School of Medicine, Kanazawa University, Kanazawa 920-8640, Japan

## Abstract

Caroli's disease belongs to a group of hepatic fibropolycystic diseases and is a hepatic manifestation of autosomal recessive polycystic kidney disease (ARPKD). It is a congenital disorder characterized by segmental saccular dilatations of the large intrahepatic bile duct and is frequently associated with congenital hepatic fibrosis (CHF). The most viable theory explaining its pathogenesis suggests that it is related to ductal plate malformation. The development of the polycystic kidney (PCK) rat, an orthologous rodent model of Caroli's disease with CHF as well as ARPKD, has allowed the molecular pathogenesis of the disease and the therapeutic options for its treatment to be examined. The relevance of the findings of studies using PCK rats and/or the cholangiocyte cell line derived from them to the pathogenesis of human Caroli's disease is currently being analyzed. Fibrocystin/polyductin, the gene product responsible for ARPKD, is normally localized to primary cilia, and defects in the fibrocystin from primary cilia are observed in PCK cholangiocytes. Ciliopathies involving PCK cholangiocytes (cholangiociliopathies) appear to be associated with decreased intracellular calcium levels and increased cAMP concentrations, causing cholangiocyte hyperproliferation, abnormal cell matrix interactions, and altered fluid secretion, which ultimately result in bile duct dilatation. This article reviews the current knowledge about the pathogenesis of Caroli's disease with CHF, particularly focusing on studies of the mechanism responsible for the biliary dysgenesis observed in PCK rats.

## 1. Introduction

Caroli's disease belongs to a group of hepatic fibropolycystic diseases [1, 2]. It is a congenital disorder characterized by a biliary abnormality consisting of segmental saccular dilatations of the large intrahepatic bile duct. It is frequently associated with varying degrees of portal fibrosis, corresponding to congenital hepatic fibrosis (CHF). Caroli initially described two variants of the biliary abnormality with and without CHF (Caroli's syndrome and Caroli's disease), and the form without CHF is quite rare. 

A significant proportion of Caroli's disease cases involving CHF are transmitted in an autosomal recessive manner and are associated with autosomal recessive polycystic kidney disease (ARPKD). The incidence of ARPKD is 1 in 20,000 live births [3]. Renal failure may be present at birth, and the disease presentation is not limited to the neonatal period; it can be diagnosed in childhood or even adolescence or adulthood [4]. These late-presenting cases typically display less severe kidney disease, but more commonly involve liver disease complications.

Caroli's disease is a developmental anomaly, and the most viable theory explaining its pathogenesis is that it is related to ductal plate malformation at different levels of the intrahepatic biliary tree [5]. Intrahepatic bile ducts develop from bipotential liver progenitor cells that are in contact with the mesenchyme of the portal vein, which form from the ductal plates [6]. The ductal plates are then remodeled into mature tubular ducts. The ductal plate remodeling process begins from the larger ducts to the smaller peripheral ducts. The heredity factors causing Caroli's disease can exert their influence not only during the early embryological period in which large intrahepatic duct formation occurs, but also during the later development of the more proximal interlobular ducts involved in CHF.

 The molecular pathogenesis of Caroli's disease is incompletely understood. Human and experimental data have suggested several potential mechanisms that could lead to cyst formation in fibropolycystic liver diseases including those of Caroli's disease patients: (i) increased cell proliferation and apoptosis; (ii) enhanced fluid secretion; (iii) abnormal cell-matrix interactions; (iv) alterations in cell polarity; and (v) abnormal ciliary structure or function [7]. To study the cyst pathogenesis of ARPKD, different experimental animal models including cpk, bpk, and orpk mice and several types of knockout mice have been used [8–12]. Among them, the polycystic kidney (PCK) rat is an orthologous model of ARPKD that represents the phenotype of the slowly progressive form of ARPKD and is also a novel animal model of Caroli's disease with CHF [13].

This article reviews our current knowledge of the pathogenesis of Caroli's disease with CHF, particularly focusing on studies about the mechanism responsible for the biliary dysgenesis observed in the PCK rat. First, the clinicopathological and genetic aspects of Caroli's disease with CHF are described. In the following section, Caroli's disease refers to the form of the disease associated with CHF, since Caroli's disease without CHF is rare.

## 2. Caroli's Disease

### 2.1. Clinical Features

Renal involvement is encountered in up to 60% of patients with Caroli's disease [14]. Hepatic manifestations of ARPKD are present in 15–45% of patients and include an enlarged liver, portal hypertension, or abnormal findings on hepatic imaging [15]. A few rare cases of Caroli's disease have occurred in the setting of autosomal dominant polycystic kidney disease (ADPKD) [16].

The clinical manifestations of Caroli's diseases are related to both biliary abnormalities and portal hypertension due to CHF [14, 17, 18]. Its clinical progression and presentation are highly variable, and symptoms may appear late in life.

Bile ducts dilatation induces a predisposition to bile stagnation, leading to the formation of lithiases. Bacterial cholangitis occurs frequently and may be complicated by hepatic abscess formation and sepsis. Recurrent cholangitis dominates the clinical course and is the principal cause of morbidity and mortality. After cholangitis occurs, a large number of patients die within 5–10 years [14]. Secondary biliary cirrhosis can occur due to biliary obstruction.

Portal hypertension due to CHF may lead to ascites and esophageal variceal hemorrhaging. Splenomegaly and hepatomegaly are common. Children with Caroli's disease usually display earlier symptom onset and a more rapidly progressive disease because of the combined effects of cholangitis and portal hypertension.

Caroli's disease may progress to cholangiocarcinoma. The occurrence of cholangiocarcinoma has been reported in 7–14% of patients [19]. A rare case of cholangiocarcinoma arising in CHF has also been reported [20].

The laboratory findings of Caroli's disease are nonspecific. Transaminase levels may be slightly elevated. A complete blood count might reveal thrombocytopenia and leukopenia if portal hypertension and hypersplenism are present. An elevated white blood cell count and increased serum alkaline phosphatase or direct bilirubin levels could indicate cholangitis. BUN and creatinine values should also be measured to detect any associated renal disease.

### 2.2. Pathology

**Figure 1:**
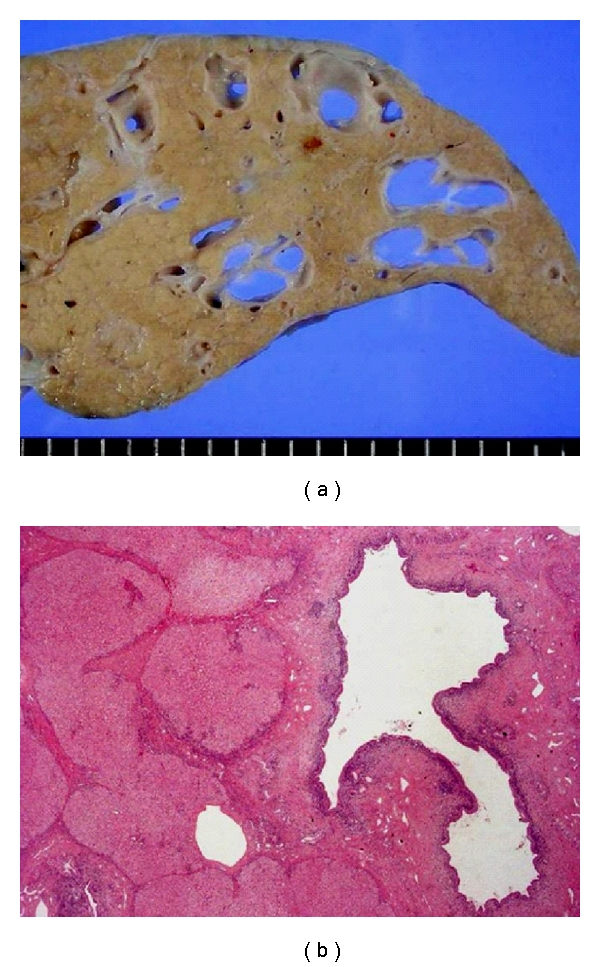
The liver of a Caroli's disease patient with CHF. Multiple cystic dilatations of the intrahepatic bile ducts are grossly (a) and histologically (b) visible. Hematoxylin-eosin staining (b).

The biliary abnormalities of Caroli's disease are characterized by progressive and segmental saccular or cystic dilatation of the intrahepatic bile duct (Figure 1). The disease might be limited to one lobe of the liver, most commonly the left lobe. Histologically, the dilated ducts are lined by the biliary epithelium, which may be hyperplastic and ulcerated. In patients with cholangitis, an acute and chronic inflammatory cell infiltrate is seen around the dilated bile ducts. In the presence of CHF, dense portal fibrosis is observed, and the fibrotic region often contains variable numbers of abnormally shaped bile ducts and hypoplastic portal vein branches. The hepatic parenchyma is subdivided by the overgrowth of portal fibrous tissue, while no parenchymal regenerative activity is evident, which allows the condition to be differentiated from cirrhotic regenerative nodules.

The mechanism of the development of cholangiocarcinoma in Caroli's disease remains unclear. In chronic biliary diseases, it has become evident that cholangiocarcinoma arising in the large bile ducts undergoes a multistep carcinogenic process, and biliary intraepithelial neoplasia (BilIN) is considered to be the precursor lesion [21]. BilIN is frequently seen in patients with hepatolithiasis, and it can be encountered in the livers of Caroli's disease patients. Biliary papillomatosis has also been observed in Caroli's disease [22]. Thus, cholangiocarcinoma in Caroli's disease probably arises despite a multistep carcinogenic process that is closely related to chronic epithelial damage.

### 2.3. Pathological Studies

There is limited data available from pathological molecular studies using human liver tissues from patients with Caroli's disease and/or CHF. Cholangiocytes from the livers of patients with Caroli's liver have been shown to overexpress vascular endothelial growth factor (VEGF), its receptors (VEGFR-1 and VEGFR-2), and angiopoietin-2 [23]. VEGF expression on cholangiocytes positively correlates with microvascular density around the bile ducts, suggesting that it has a proliferative effect on cholangiocyte growth by inducing the production of an abundant vascular supply. VEGF may also stimulate bile duct dilatation through the induction of cholangiocyte proliferation via an autocrine effect [24]. In addition, the activation of the mammalian target of rapamycin (mTOR) pathway has been implicated in the overgrowth of cholangiocytes in Caroli's disease [25].

Cholangiocyte overgrowth is linked to abnormalities in cell cycle progression and also to microRNA expression. The progression of cells through the cell cycle is controlled by a family of dual specificity phosphatases, Cdc25, that activate cyclin-dependent kinases. The biliary epithelium of CHF overexpresses Cdc25A protein (an isoform of Cdc25), which is accompanied by the downregulation of a microRNA (miR15a) [26]. 

Around intrahepatic bile ducts, basement membrane components such as laminin and type IV collagen, the major basal laminar components are degraded in Caroli's disease [27]. These findings indicate that the reduction of laminin and type IV collagen expression in the basement membrane, a supportive structure of intrahepatic bile ducts exacerbates the observed bile duct dilatation. The degradation of laminin and type IV collagen around bile ducts is also observed in foci of cholangiocarcinoma in situ arising in Caroli's disease, indicating that once cholangiocarcinoma in situ develops in the biliary epithelia of Caroli's disease patients, it tends to transform into invasive carcinoma [27].

 In most types of chronic liver disease, activated hepatic stellate cells play major roles in hepatic fibrosis. However, necroinflammatory changes and the activation of hepatic stellate cells are not as marked in CHF as those seen in ordinary chronic liver diseases such as chronic viral hepatitis. The fact that abundant connective tissue growth factor (CTGF) is retained by heparin sulfate proteoglycans (HSPG) in the fibrous portal tracts could be responsible for the unresolved hepatic fibrosis observed in CHF [28]. Portal mononuclear cells and endothelial cells expressing CTGF and/or HSPG tend to collect around proliferated bile ducts in CHF, providing a possible explanation for the mechanism of the fibrosis that characterizes CHF.

### 2.4. Diagnosis

**Figure 2:**
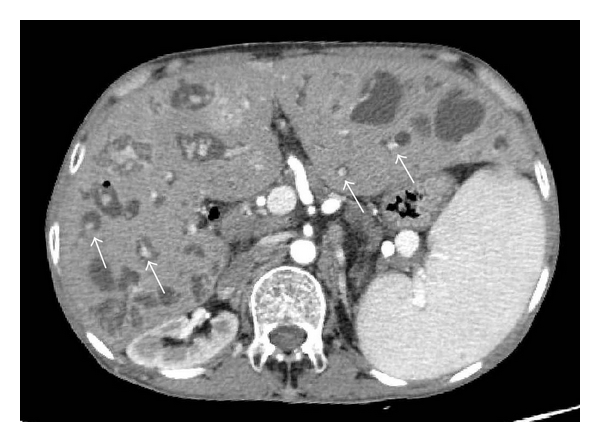
Dynamic CT reveals multiple cystic dilatations of the intrahepatic bile ducts in a patient with Caroli's disease with CHF. The arrows indicate the central dot sign.

Caroli's disease is diagnosed by imaging studies showing nonobstructive, saccular, or fusiform dilatations of the intrahepatic bile ducts [29–31]. Ultrasonography and endoscopic retrograde cholangiopancreatography are the traditional methods of diagnosis. However, magnetic resonance cholangiopancreatography is emerging as the most useful diagnostic modality. On sonography, Caroli's disease presents as intrahepatic cystic anechoic areas in which fibrovascular bundles and linear bridges or septa may be present. The fibrovascular bundles are composed of the portal veins and hepatic arteries, which can be demonstrated by Doppler ultrasonography and are recognized as the central dot sign on CT with contrast enhancement (Figure 2). Overlapping imaging findings are often detected, which reflect its underlying pathology and associated complications, including fibrosis, ductal dilatation, cholangitis, lithiasis, and malignancy [32]. A liver biopsy is rarely required to make a diagnosis of Caroli's disease.

### 2.5. Treatment

Treatment for Caroli's disease is largely supportive and is directed toward treating the biliary infection and complications associated with portal hypertension [15]. Cholangitis, hepatic abscesses, and sepsis should be treated aggressively with appropriate antibiotics. Infections are particularly difficult to eradicate in the presence of bile stasis and intrahepatic lithiasis. Recurrent bouts of cholangitis can lead to end-stage liver disease. 

Common bile duct stones may require endoscopic sphincterotomy and stone extraction, while the extraction of intrahepatic stones is difficult. Partial hepatectomy may be curative when the disease is confined to a single lobe of the liver [33]. Ursodeoxycholic acid has been used to treat intrahepatic lithiasis, which probably acts by increasing bile flow and decreasing bile stasis [34].

Variceal bleeding can be treated endoscopically with sclerotherapy or band ligation. A selective shunting procedure can provide relief from the complications associated with portal hypertension.

Liver transplantation is regarded as the ultimate treatment for patients who suffer recurrent bouts of biliary infection and those who also have complications related to portal hypertension [35]. Patient survival is reported to be excellent, and graft survival is comparable to or better than that of patients who have received transplants for other diseases [36]. Since the inheritance of the disease seems to occur in an autosomal recessive manner, it is important to provide genetic counseling to the patient's family.

### 2.6. Genetics

ARPKD is caused by mutations in a single gene, *PKHD1*, which has been localized to chromosome 6p21.1-p12. The gene consists of 86 exons and has a number of alternatively spliced transcripts. Its longest open reading frame contains 67 exons, which encode a 4074 amino acid protein called fibrocystin or polyductin [37, 38]. 

*PKHD1* exhibits a high level of allelic heterogeneity, and more than 300 mutations have been described throughout *PKHD1*. A clear genotype/phenotype correlation has been described in ARPKD, with two truncating mutations associated with the most severe phenotype, while one or two missense changes are associated with milder disease [39]. The genetic basis for the differences between ARPKD with and without CHF has not been fully elucidated [40]. Mutations in *PKHD1* have also been identified in patients with Caroli's disease. *PKHDL1*, a homologous gene that is not involved in renal cystic disease has also been described [41].

### 2.7. Fibrocystin/Polyductin

Fibrocystin is a receptor-like membrane-associated protein. Structural predictions indicate that it has a large extracellular region with multiple copies of the TIG domain (an immunoglobulin-like fold), a single transmembrane region, and a short cytoplasmic tail. Based on its similarity with other TIG-containing proteins such as the hepatocyte growth factor receptor MET, fibrocystin is suggested to function as a receptor or ligand, since secreted forms can be generated from alternatively spliced transcripts [42]. In addition, the promoter might be directly regulated by hepatocyte nuclear factor-1*β* [43].

*PKHD1* is preferably expressed in the kidneys with lower levels observed in the liver, pancreas, and lungs [37]. Similarly, fibrocystin is expressed in the cortical and medullary collecting ducts of the kidney as well as the biliary and pancreatic ducts [44], in which its distribution is consistent with the disease's phenotype. Fibrocystin is localized to primary cilia as well as to the basal body of epithelial cells and colocalizes with polycystin-2, the gene product responsible for ADPKD [42, 45]. Fibrocystin is also expressed in the normal ductal plate as well as in ductal plate malformations including CHF and colocalizes with stem cell markers in some ductal plate cells [46]. 

Fibrocystin can undergo notch-like processing, resulting in the release of the ectodomain from primary cilia [47]. Other studies have demonstrated cleavage of the fibrocystin ectodomain as well as the generation of a cytoplasmic fragment that translocates to the nucleus [48]. Such proteolytic cleavage can be elicited by the stimulation of intracellular calcium release or protein kinase C activation. Fibrocystin and polycystin-2 may act in a common molecular pathway to regulate calcium responses in the epithelia [49]. The structure and homologies of fibrocystin suggest that it plays a role in the regulation of cellular adhesion, repulsion, and proliferation and/or the regulation and maintenance of renal collecting tubules and bile ducts, but its exact role in normal and cystic epithelia remains unknown.

## 3. The PCK Rat

The PCK rat is derived from a Crj:CD (Sprague-Dawley) rat strain, originating in Japan [50]. The polycystic disease it suffers from is inherited in an autosomal recessive manner, and this model has a spontaneous mutation in its *Pkhd1* gene, an ortholog of human *PKHD1* [37]. The model has been rederived and is commercially available from Charles River Laboratories (Wilmington, MA).

**Figure 3:**
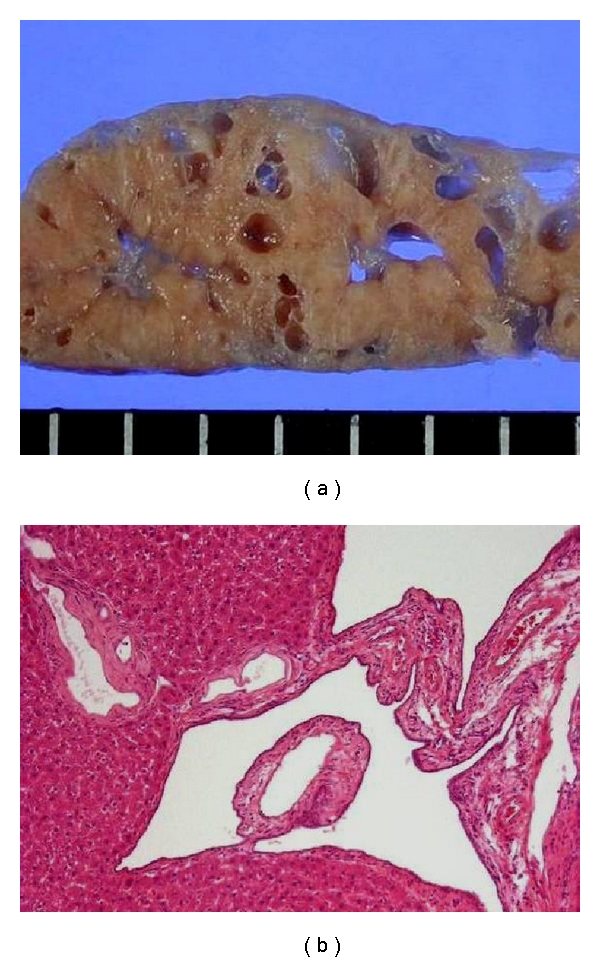
The liver of a PCK rat. The gross (a) and histological (b) appearance of the adult rat liver closely resembles those of patients with Caroli's disease with CHF (Figure 1). Hematoxylin-eosin staining (b).

**Figure 4:**
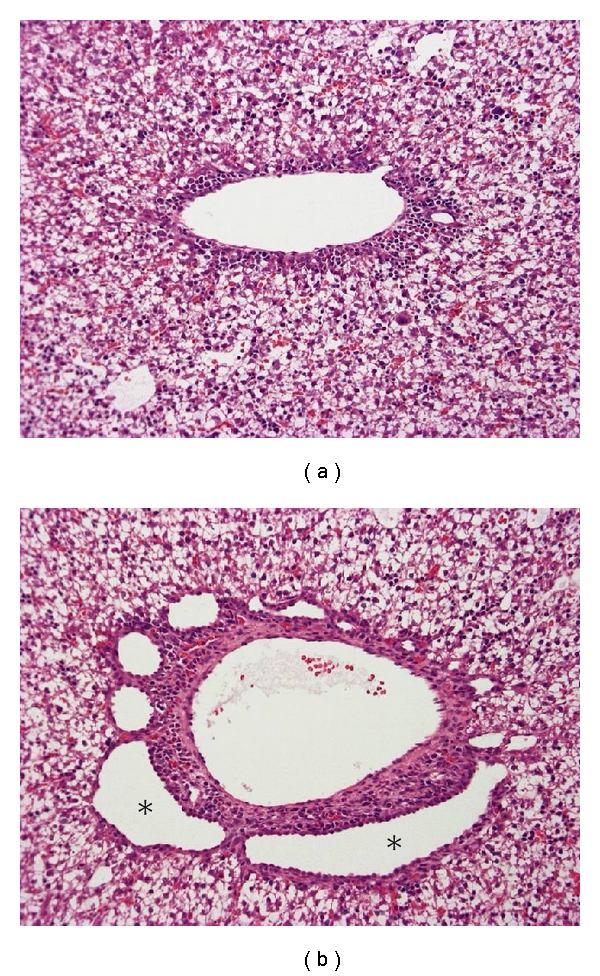
Ductal plate malformation in the fetal liver of a PCK rat. Compared with a normal fetal rat (a), dilatation of the ductal plate is evident in the PCK liver (b, asterisks). Hematoxylin-eosin staining (a, b).

In the livers of PCK rats, multiple segmental and saccular dilatations of the intrahepatic bile duct are observed (Figure 3). In addition, ductal plate malformations are evident in the livers of PCK rat fetuses (Figure 4), and the ductal dilatation spreads throughout the liver and increases in degree with age. The overgrowth of portal connective tissue progresses after delivery. All of the gross and histological features of the PCK liver correspond to Caroli's disease with CHF. 

To explore the mechanism of biliary dysgenesis suffered by PCK rats, a cholangiocyte cell line has been developed from the intrahepatic bile ducts of the PCK rat [51, 52]. The cholangiocytes of the PCK rat exhibit a higher rate of proliferation, with a doubling time of approximately half that of normal cholangiocytes, and maintain their biliary features during passaging. 

Hereafter, based on the results of studies using PCK rats and/or the cholangiocyte cell line derived from them, the recent developments in our understanding of the cellular and molecular pathogeneses of the biliary dysgenesis observed in PCK rats are reviewed. 

### 3.1. Proliferation and Apoptosis

Two key signaling pathways, 3′,5′-cyclic adenosine monophosphate (cAMP) activated B-Raf/MEK/ERK and AKT/mTOR/S6K/S6, have been implicated in the increased proliferation of PCK cholangiocytes.

Epidermal growth factor (EGF) and its receptor (EGFR) play important roles in promoting cholangiocyte proliferation. PCK cholangiocytes are hyperresponsive to EGF, and the increase in their proliferation is accompanied by activation of the MEK5/ERK5 pathway [51]. The phosphorylation of ERK1/2 is also increased in PCK cholangiocytes [53]. In contrast to other mouse models of ARPKD, EGFR is not overexpressed or mislocalized to the apical membrane in the PCK cholangiocytes [51, 54].

The cAMP levels of the PCK cholangiocytes are also increased [55]. These elevated cAMP levels stimulate cholangiocyte proliferation via downstream effectors and exchange proteins activated by cAMP (Epac1 and Epac2 isoforms) and protein kinase A (PKA) [56]. Hyperproliferation of the PCK cholangiocytes in response to PKA stimulation is associated with decreased intracellular calcium levels, and the restoration of calcium levels blocks PKA-dependent proliferation via the PI3K/AKT pathway. In addition, PCK cholangiocyte hyperproliferation is accompanied by the overexpression of Cdc25A protein and the downregulation of miR15a [26, 57]. miR15a overexpression in PCK cholangiocytes decreases Cdc25A levels, inhibits cell proliferation, and reduces cyst growth, indicating a potential therapeutic strategy for the disease.

The signaling pathway composed of AKT/mTOR/S6K/S6 is activated in PCK cholangiocytes (Ren XS et al., unpublished data). In the PCK rat liver, apoptosis of the biliary epithelium is less frequent than in normal rats until 1 week after delivery but is more common than in normal rats at 3 weeks after delivery [13]. Thus, dysregulated cell kinetics may be involved in the biliary abnormalities associated with PCK rats.

### 3.2. Fluid Secretion

Activated cAMP pathways can also lead to increased fluid secretion. Indeed, bile secretion is increased in the PCK rats compared with that in age-matched normal rats [58]. In normal cholangiocytes, the water channel aquaporin-1, the chloride channel cystic fibrosis transmembrane conductance regulator (CFTR), and the anion exchanger AE2 regulate ion-driven water transport. In PCK cholangiocytes, aquaporin-1, CFTR, and AE2 are overexpressed and show abnormal cellular localization, which could account for their altered fluid secretion [59].

### 3.3. Cell-Matrix Interactions

As is true in human Caroli's disease, the matrix proteins of the basement membranes of the intrahepatic bile ducts are degraded in PCK rats, and the biliary epithelium sits on the basement membrane and displays abnormal decreases in laminin and type IV collagen expression [27]. Since PCK cholangiocytes overexpress plasminogen and the tissue-type plasminogen activator, the generation of excessive amounts of plasmin and the subsequent plasmin-dependent lysis of extracellular matrix molecules may contribute to the progressive bile duct dilatation observed.

### 3.4. Primary Cilia and Ciliopathies

Cholangiocytes are ciliated cells, and cholangiocyte cilia extend from the apical plasma membrane into the bile duct lumen [60]. Cholangiocyte primary cilia are mechanosensory, osmosensory, and chemosensory organelles that can detect changes in bile flow and osmolarity and transduce them into intracellular signals. Changes in flow are communicated to other cellular response elements via changes in intracellular calcium and cAMP concentrations. Increased flow causes a cilium-dependent rise in intracellular calcium followed by a decrease in the cAMP concentration via a calcium-inhibitable adenyl cyclase.

In PCK rats, a splicing mutation in *Pkhd1* results in structural and functional ciliary abnormalities [61]. Fibrocystin is normally localized to primary cilia, whereas defects in fibrocystin from primary cilia are observed in PCK cholangiocytes [62]. Defects in ciliary structure and their integrated sensory/transducing functions appear to result in decreased intracellular calcium and increased cAMP concentrations, causing cholangiocyte hyperproliferation, abnormal cell-matrix interactions, and altered fluid secretion. These modifications can lead to abnormalities in biliary tree differentiation, ultimately resulting in bile duct dilatation. 

Other calcium channels such as Trpv4 are present in cholangiocyte cilia, and the activation of Trpv4 leads to increased intracellular calcium levels and reduces the hyperproliferative phenotype of PCK cholangiocytes [63].

In the liver, mutations in genes encoding ciliary-associated proteins cause a broad spectrum of genetically heterogeneous disorders, which are referred to as ciliopathies [64]. Since cholangiocytes are the only epithelial cells in the liver that possesses primary cilia, conditions affecting the liver are more appropriately called cholangiociliopathies [65].

### 3.5. Cholangitis

As PCK rats age, chronic suppurative cholangitis becomes a frequent histologic finding [66]. Although the clinical significance of cholangitis due to biliary infection is well recognized in Caroli's disease, the impact of biliary infection on its pathogenesis and progression is poorly understood.

Lipopolysaccharides (LPS) induce VEGF expression in PCK cholangiocytes via toll-like receptor 4 expressed on the cells, which is accompanied by the activation of NF-*κ*B (Ren XS et al., unpublished data). Both LPS and VEGF increase the proliferation of PCK cholangiocytes, suggesting that LPS-induced overexpression of VEGF in the biliary epithelium leads to hypervascularity around the bile ducts, and concurrently, LPS and VEGF act as cell proliferative factors for cholangiocytes. Thus, biliary infection may exacerbate biliary cystogenesis through the induction of VEGF in the biliary epithelia of PCK rats.

Cholangitis is frequently associated with goblet cell metaplasia of the biliary epithelium in PCK rats. LPS induces upregulated CDX2 expression followed by aberrant mucus core protein-2 expression via the activation of NF-*κ*B in PCK cholangiocytes, which accounts for the development of intestinal metaplasia in the setting of biliary infection [66]. Although recurrent cholangitis probably leads to the development of cholangiocarcinoma in some patients with Caroli's disease, we have not encountered the occurrence of cholangiocarcinoma in PCK rats.

### 3.6. Hepatic Fibrosis

A mechanism similar to the epithelial-mesenchymal transition (EMT) has been implicated in the hepatic fibrosis observed in PCK rats [67]. In PCK rat liver sections, the intrahepatic bile ducts display two different phenotypes, bile ducts lined by cuboidal-shaped (C-type), and flat-shaped (F-type) cholangiocytes. The flat-shaped cholangiocytes (F-type) show reduced immunohistochemical expression of the biliary epithelial marker cytokeratin19 and positive immunoreactivity for the mesenchymal markers vimentin and fibronectin. Treating PCK cholangiocytes with transforming growth factor-*β*1 (TGF-*β*1), a potent inducer of EMT induces the expression of vimentin and fibronectin in vitro, indicating that PCK cholangiocytes acquire mesenchymal features in response to TGF-*β*1 and participate in progressive hepatic fibrosis by producing extracellular matrix molecules. EMT has been also implicated in the pathogenesis of interstitial fibrosis in the kidneys of PCK rats [68].

In elderly PCK rats, suppurative cholangitis is a frequent histological finding in C-type cholangiocytes, while F-type cholangiocytes are not associated with suppurative cholangitis accompanied by polymorphonuclear leukocyte accumulation in their lumen [67]. In addition, F-type cholangiocytes occasionally show a fibrous scar-like appearance. Recent studies have shown that the majority of dilated intrahepatic bile ducts in PCK rats are initially connected to the biliary tree but over time become separated from it, resulting in true cyst formation [61]. It is speculated that F-type cholangiocytes are derived from the bile ducts that have been disconnected from the biliary tree, which may account for the observation of true biliary cysts in PCK rats.

The renin-angiotensin system is upregulated in the livers of PCK rats [69]. Angiotensin-converting enzyme (ACE) and angiotensin II, as well as their downstream target, the profibrotic mediator TGF-*β*, are overexpressed in the PCK liver, suggesting that the renin-angiotensin system activation is another important mediator of hepatic fibrosis.

### 3.7. Therapeutic Approaches

Understanding of the molecular mechanisms of cyst formation and growth has led to the discovery of novel potential therapeutic approaches for fibropolycystic diseases. However, in PCK rats, relatively few therapeutic reagents are effective for both liver and kidney cystogenesis.

Octreotide, a somatostatin analogue known to inhibit cAMP, decreases hepatic cyst volume, the hepatic fibrosis score, and mitotic indices in the PCK liver, and similar effects are observed in the kidneys [55]. Pioglitazone, a peroxisome proliferator activator receptor gamma agonist, inhibits bile duct dilatation and hepatic fibrosis as well as renal cyst growth, which is associated with decreased CFTR expression and reduced cell proliferation [53, 70].

As another example of therapies that are effective for both liver and kidney lesions in PCK rats, the inhibition of Src activity with SKI-606 ameliorates biliary ductal abnormalities and renal cyst formation [71]. The effects of Src inhibition suggest that the Erb2 and B-Raf/MEK/ERK pathways are involved in Src mediated signaling in ARPKD and that this occurs without any reduction in cAMP levels.

The inhibition of renal cAMP production by treatment with a vasopressin V2 receptor antagonist or by increasing water intake to reduce plasma vasopressin decreases cell proliferation and ameliorates kidney cystogenesis with an associated reduction in B-Raf/MEK/ERK activity, leading to improved renal function in PCK rats [72–74]. However, consistent with the absence of the vasopressin V2 receptor in the liver, it does not have a significant effect on fibropolycystic liver disease. Similarly, Trpv4 activation induces a significant decrease in renal cystic area but causes a nonsignificant decrease in liver cyst formation [63]. 

ACE inhibition by chronic treatment with lisinopril decreases proliferative and apoptotic pathways in the kidneys of PCK rats, resulting in improved kidney function [75]. Chronic blockade of 20-hydroxyeicosatetraenoic acid (HETE) with a specific inhibitor of the CYP4A and CYP4F enzyme family prevents the formation of 20-HETE, resulting in a significant decrease in renal cyst formation in PCK rats [76]. The activation of calcium-sensing receptors with R-568 reduces the interstitial fibrosis, but not the cystogenesis, of the PCK kidney [77]. However, it is unclear whether these treatments, that is, the inhibition of ACE and 20-HETE synthesis, and the activation of calcium-sensing receptors, are effective treatments for biliary dysgenesis in PCK rats.

Gefitinib, an EGFR tyrosine kinase inhibitor, significantly improves biliary cystogenesis and hepatic fibrosis in PCK rats but has no beneficial effects on renal cyst pathogenesis [78]. In addition, EGFR tyrosine kinase inhibition with EKI-785 and EKB-569 has no effect on biliary dysgenesis in PCK rats, and the kidney lesions are unaffected or rather worsened by the treatment [54]. The inhibition of mTOR with sirolimus does not attenuate the progression of liver or kidney disease in PCK rats, which may be due to intrinsic or acquired sirolimus resistance [79].

## 4. Conclusions

The development of PCK rats has allowed us to explore the molecular pathogenesis of the disease and potential therapeutic strategies for Caroli's disease and ARPKD. The relevance of findings obtained from studies using PCK rats and/or the cholangiocyte cell line derived from them to the pathogenesis of human diseases is currently being analyzed, and several key signaling pathways have been elucidated. It seems likely that future treatments for Caroli's disease will involve combination therapies affecting several cystogenesis pathways.

## Abbreviations

ACE: Angiotensin-converting enzyme
ADPKD: Autosomal dominant polycystic kidney disease
ARPKD: Autosomal recessive polycystic kidney disease
BilIN: Biliary intraepithelial neoplasia
cAMP: 3′,5′-Cyclic adenosine monophosphate
CHF: Congenital hepatic fibrosis
CFTR: Cystic fibrosis transmembrane conductance regulator
CTGF: Connective tissue growth factor
EGF: Epidermal growth factor
EGFR: EGF receptor
EMT: Epithelial-mesenchymal transition
HETE: Hydroxyeicosatetraenoic acid
HSPG: Heparin sulfate proteoglycan
LPS: Lipopolysaccharide
mTOR: Mammalian target of rapamycin
PKA: Protein kinase A
PCK: Polycystic kidney
TGF-*β*: Transforming growth factor-*β*
VEGF: Vascular endothelial growth factor
VEGFR: VEGF receptor.
